# Targeted therapy by combined inhibition of the RAF and mTOR kinases in malignant spindle cell neoplasm harboring the *KIAA1549-BRAF* fusion protein

**DOI:** 10.1186/1756-8722-7-8

**Published:** 2014-01-14

**Authors:** Vivek Subbiah, Shannon N Westin, Kai Wang, Dejka Araujo, Wei-Lien Wang, Vincent A Miller, Jeffrey S Ross, Phillip J Stephens, Gary A Palmer, Siraj M Ali

**Affiliations:** 1 Department of Investigational Cancer Therapeutics (Phase I Clinical Trials Program), Division of Cancer Medicine, The University of Texas MD Anderson Cancer Center, 1515 Holcombe Blvd., FC8.3038, Box 0455, Houston, TX 77030, USA; 2 Foundation Medicine, Cambridge, Massachusetts, USA; 3 Department of Sarcoma Medical Oncology, Division of Cancer Medicine, The University of Texas MD Anderson Cancer Center, 1515 Holcombe Blvd, Houston, TX 77030, USA; 4 Division of Pathology, The University of Texas MD Anderson Cancer Center, 1515 Holcombe Blvd, Houston, TX 77030, USA; 5 Division of Gynecologic Oncology and Reproductive Medicine, The University of Texas MD Anderson Cancer Center, 1515 Holcombe Blvd, Houston, TX 77030, USA; 6 Department of Pathology, Albany Medical Center, Albany, NY 12222, USA

**Keywords:** Spindle cell neoplasm, Sarcoma, *KIAA1549-BRAF*, BRAF, Targeted therapy, Sorafenib, mTOR, Bevacizumab, Next generation sequencing, N = 1 trial

## Abstract

**Background:**

Oncologic patients who are extreme responders to molecularly targeted therapy provide an important opportunity to better understand the biologic basis of response and, in turn, inform clinical decision making. Malignant neoplasms with an uncertain histologic and immunohistochemical characterization present challenges both on initial diagnostic workups and then later in management, as current treatment algorithms are based on a morphologic diagnosis. Herein, we report a case of a difficult to characterize sarcoma-like lesion for which genomic profiling with clinical next generation sequencing (NGS) identified the molecular underpinnings of arrested progression(stable disease) under combination targeted therapy within a phase I clinical trial.

**Methods:**

Genomic profiling with clinical next generation sequencing was performed on the FoundationOne™ platform (Foundation Medicine, Cambridge MA). Histopathology and immunohistochemical studies were performed in the Department of Pathology, MD Anderson Cancer Center (Houston, TX). Treatment was administered in the context of a phase I clinical trial ClinicalTrials.gov Identifier: (NCT01187199).

**Results:**

The histology of the tumor was that of a spindle cell neoplasm, grade 2 by FNCLCC standards. Immunohistochemical staining was positive for S100 and CD34. Genomic profiling identified the following alterations: a *KIAA1549-BRAF* gene fusion resulting from a tandem duplication event, a homozygous deletion of *PTEN,* and frameshift insertion/deletions in *CDKN2A* A68fs*51, *SUFU* E283fs*3, and *MAP3K1* N325fs*3. The patient had a 25% reduction in tumor (RECIST v1.1) following combination therapy consisting of sorafenib, temsirolimus, and bevazicumab within a phase I clinical trial.

**Conclusions:**

The patient responded to combination targeted therapy that fortuitously targeted *KIAA1549-BRAF* and *PTEN* loss within a spindle cell neoplasm, as revealed by genomic profiling based on NGS. This is the first report of a tumor driven by a *KIAA1549-BRAF* fusion responding to sorafenib-based combination therapy.

## Introduction

Tumors that defy ready histopathologic characterization are difficult to rationally approach in the setting of treatment algorithms which are based on the anatomic origin of the neoplasm. Spindle cell neoplasms are such tumors that defy characterization even with ancillary methods such as immunohistochemistry. In such cases, genomic profiling based on next generation sequencing (NGS) can identify the genomic alterations (GA) that drive oncogenesis and thus prospectively suggest aberrantly activated pathways for pharmacologic inhibition independent of tumor site origin. Conversely, in the case of exceptional responders to targeted therapy, diagnostic genomic profiling may uncover the linked underlying alterations retrospectively. We report one such case of a patient with a malignant spindle cell neoplasm who exhibited clinical and radiographic improvement with simultaneous RAF kinase inhibition (sorafenib/Nexavar®), mTOR inhibition (temsirolimus/Torisel®) and VEGF (bevacizumab/Avastin®) targeted therapy who had been refractory to standard cyototoxic chemotherapy. A Clinical Laboratory Improvement Amendments (CLIA) laboratory performed NGS-based diagnostic genomic profiling which identified this tumor as the first reported case of the *KIAA1549-BRAF* fusion in a PTEN null background as a driving genomic alteration susceptible to targeted therapy.

## Patients and methods

### Patient selection and clinical assessments

We reviewed the medical records of a patient with spindle cell neoplasm who presented to the Department of Investigational Cancer Therapeutics at The University of Texas MD Anderson Cancer Center after failing standard of care therapy. Treatment and consent on investigational trial, and data collection were performed in accordance with the guidelines of the University of Texas MD Anderson Cancer Center Institutional Review Board (IRB). Tumor response was determined using RECIST (version 1.1) by CT scans obtained about every six to eight weeks. Clinical evaluation and assessments were performed per protocol.

### Genomic profiling

Next-generation sequencing was performed by using the Clinical Laboratory Improvement Amendments (CLIA)-approved FoundationOne™ platform (Foundation Medicine, Cambridge, MA, USA). FoundationOne™ is a targeted assay utilizing next generation sequencing in routine cancer specimens. The assay simultaneously sequences the entire coding sequence of 236 cancer-related genes (3,769 exons) plus 47 introns of 19 genes frequently rearranged in cancer to a minimum coverage depth of 250X. The assay detects all class of genomic alterations (including base substitutions, insertions and deletions, copy number alterations and rearrangements) using routine FFPE tissue samples that may be as small as 0.6 mm^3^.

## Results and discussion

### Case history

A 55 year old female presented to the clinical center for targeted therapy to discuss treatment options for a progressive metastatic spindle cell neoplasm. Disease at presentation included a left chest wall mass measuring more than 6 cm in greatest dimension. Extent of disease evaluation also revealed a lytic lesion in the left seventh rib and a second smaller mass centered in the pleura.

**Figure 1:**
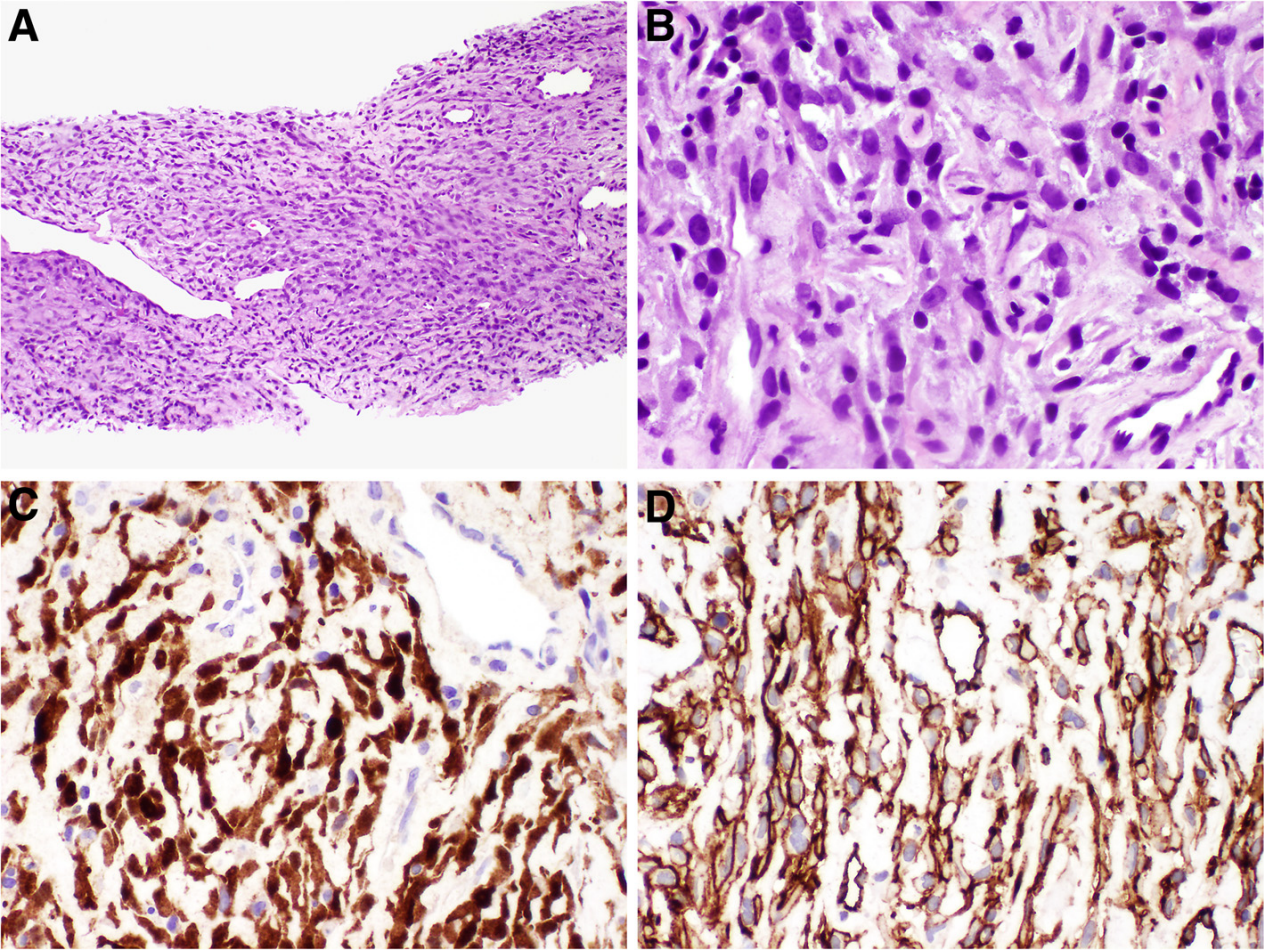
**Histopathologic examination of formalin fixed paraffin embedded (FFPE) biopsied tissue from the presumed primary tumor site. (A)** Low power (100x). Cellular tumor composed of spindled cells. **(B)** High power (400x). Short atypical spindle cells with few mitotic figures. Immunohistochemical studies reveal the tumor to be reactive for **(C)** S-100 protein and **(D)** CD34.

Pathologic examination of formalin fixed paraffin embedded (FFPE) biopsied tissue from the presumed primary tumor site revealed a spindle cell proliferation, which was diagnosed to be a malignant spindle cell neoplasm, favor sarcoma, which is akin to a diagnosis of exclusion. The diagnosis of a malignant solitary fibrous tumor was also entertained, but the features were not typical for such a diagnosis (Figure 1 A,B upper left and right panels with 100× and 400× magnification, respectively). Immunohistochemical stains for S-100 and CD34 were positive (Figure 1 C,D lower left and right panels, respectively). Notably, mitoses were counted at 6/10 per high powered field (HPF), and no necrosis was identified. Using FNCLCC guidelines for the histopathologic grading of soft tissue sarcomas as a reference, this difficult to characterize neoplasm would be intermediate grade [1].

The patient was initially treated with doxorubicin (Adriamycin**®**) 75 mg/m^2^ and ifosfamide (Ifex**®**) 10 g/m^2^. She progressed during two cycles of this treatment, and was then started on gemcitabine (Gemzar**®**) 900 mg/m^2^ and docetaxel (Taxotere**®**) 100 mg/m^2^. The patient then progressed after two cycles of her second regimen.

**Figure 2:**
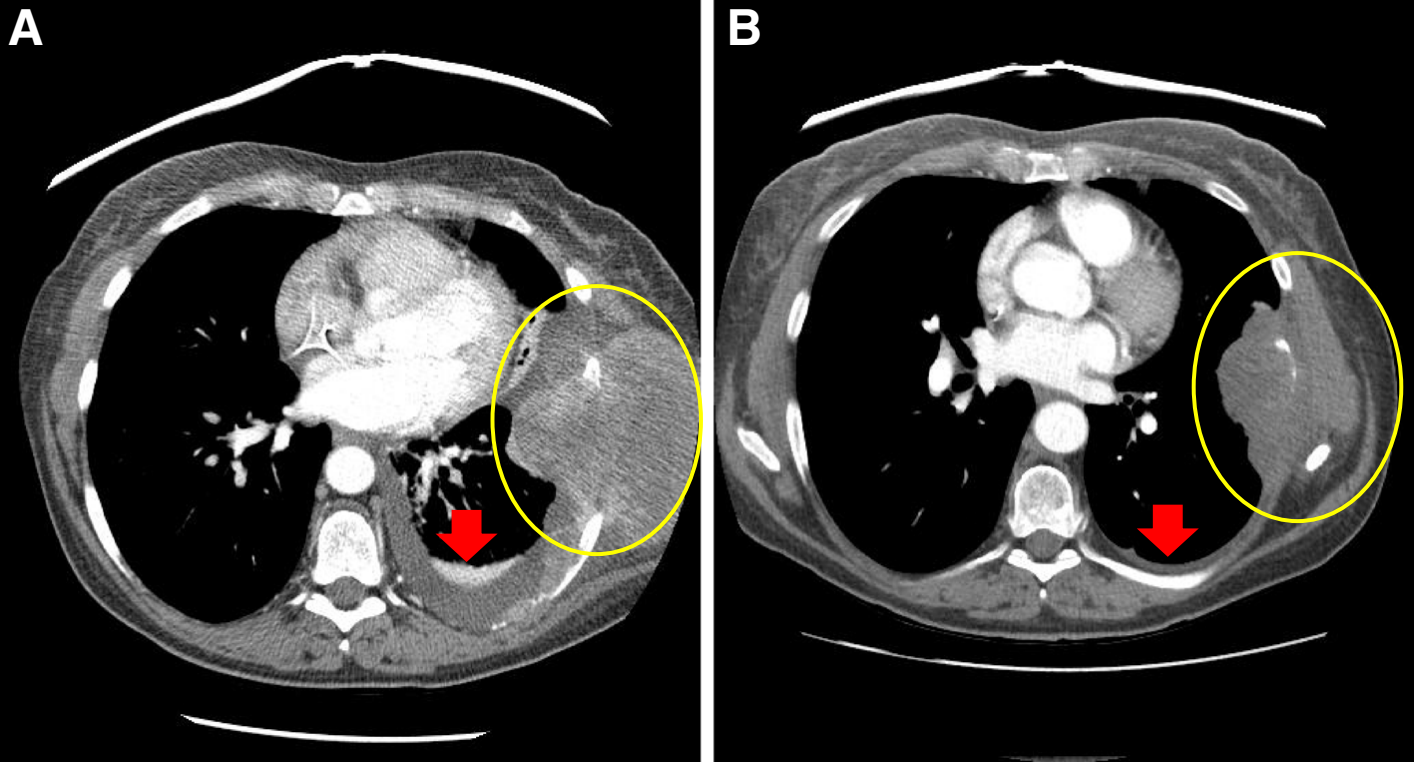
**Imaging studies pre and post- treatment. A**: Pre-treatment CT scan of the Chest shows left chest wall tumor invading the ribs and left sided pleural effusion. **B**. Post-treatment CT scan of the Chest shows a decrease in size of the tumor and decrease in pleural effusion.

The patient was then enrolled in a phase I clinical trial (ClinicalTrials.gov Identifier: NCT01187199) of bevacizumab and temsirolimus in combination with sorafenib for the treatment of advanced cancer [2]. The patient was treated with bevacizumab 10 mg/kg intravenously (IV) every 21 days, temsirolimus 20 mg IV on Day 1, 8 and 15, and sorafenib 200 mg orally twice daily. After of two cycles of therapy, the patient had a 25% reduction in greatest unidimensional tumor measurment per RECIST 1.1 (Figure 2 A,B), which is stable disease (SD), and also just below the criteria for a partial response (PR). Pain secondary to the chest wall mass decreased and dyspnea, likely secondary to the resolving pleural effusion, lessened. She tolerated therapy well, except for experiencing grade 3 hypertension related to bevacizumab that required anti-hypertensive therapy. She also had grade 2–3 hand-foot-syndrome related to sorafenib, leading to a dose reduction to 200 mg once daily. The patient continued on therapy for seven months since the initiation of therapy.

Contemporaneously with the entrance of patient into the clinical trial, FFPE tumor cell-predominant tissue from the biopsy was submitted to a commercial CLIA-certified, CAP-accredited laboratory for genomic profiling based on next generation sequencing (NGS) based.

At the time of this report, the patient’s overall response was stable disease for eleven cycles. She had the following ongoing toxicities that were felt to be at least possibly related to the study drug: grade 1 altered taste, grade 2 proteinuria, grade 1 hand-foot syndrome, grade 2 hypoalbuminemia, and grade 2 mylagias. The patient did not experience a dose limiting toxicity from the combination therapy. Unfortunately, the patient developed an acute myocardial infarction, hypotension, clinically deteriorated and died because of this co-morbidity.

### Genomic profiling

**Figure 3:**
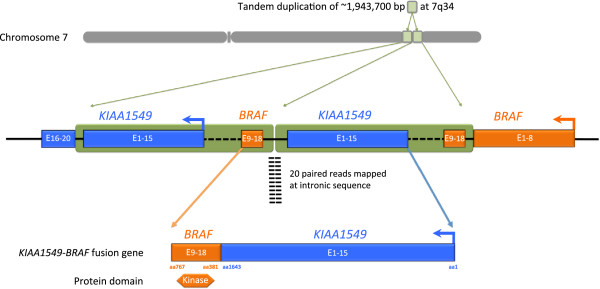
***KIAA1549-BRAF *fusion protein.** Of the two components of the *KIAA1549-BRAF* fusion protein, BRAF is a cytosolic serine/threonine kinase that is activated by binding of RAS-GTP. The function of *KIAA1549* remains unknown. This fusion merges intron 15 of *KIAA1549* and intron 8 of *BRAF*, although several other configurations of these fused genes have been observed. The kinase domain and ATP binding pocket of BRAF are retained but the N-terminal conserved region 1 (CR1) and conserved region 2 (CR2) are lost. CR1 auto-inhibits the kinase domain, but this inhibitory allosteric interaction is disrupted by the binding of RAS-GTP to CR1. Thus, loss of the CR1 domain suggests a dysregulation of the kinase activity of this fusion.

3769 exons of 236 cancer-related genes and 47 introns from 19 genes frequently rearranged in cancer were sequenced to a depth of coverage of 835×. A tandem duplication predicted to generate a *KIAA1549-BRAF* fusion gene (Figure 3), was identified as well as a homozygous deletion of *PTEN*. Other genomic alterations identified in this tumor were frameshift insertion/deletions in *CDKN2A* A68fs*51, *SUFU* E283fs*3, and *MAP3K1* N325fs*3.

## Discussion

*KIAA1549-BRAF* is a recurrent oncogenic driver in sporadic pilocytic astrocytoma, but is described here in a spindle cell neoplasm for the first time [3]. To our knowledge, moreover, this case is the first report any tumor expressing this fusion to be successfully treated by targeted therapy. The patient initially received two lines of standard chemotherapy for soft tissue sarcoma, and did not respond. For this patient, the administered combination of sorafenib, temsirolimus, and bevacizumab provided durable disease control and symptomatic benefit. Likely, the magnitude and rapidity of this response was due to treatment that precisely and fortuitously targeted the genomic alterations present in this tumor.

A possible pathologic diagnosis for this tumor could be that of a malignant peripheral nerve sheath tumor (MPNST) due to the positive S100 immunohistochemical stain and histologic phenotype of spindle cells, although that diagnosis was not made here. Interestingly, the known activating V600E *BRAF* mutation is observed in a non-syndromic and sporadic subset of MPNST cases [4]. The identification of a *BRAF* fusion in this spindle cell tumor is consistent with molecular and phenotypic correlation of an activated *BRAF* in some MPNST cases.

Of the two components of the *KIAA1549-BRAF* fusion protein, BRAF is a cytosolic serine/threonine kinase that is activated by binding of RAS-GTP. The function of *KIAA1549* remains unknown. This fusion joins intron 15 of *KIAA1549* and intron 8 of *BRAF*, although several other configurations of these fused genes have been observed [5]. The kinase domain and ATP binding pocket of BRAF are retained but the N-terminal conserved region 1 (CR1) and conserved region 2 (CR2) are lost. CR1 auto-inhibits the kinase domain, but this inhibitory allosteric interaction is disrupted by the binding of RAS-GTP to CR1. Thus, loss of the CR1 domain suggests a dysregulation of the kinase activity of this fusion.

Sorafenib is a small molecule inhibitor of WT BRAF and other kinases including VEGFR 1/2/3, PDGFRB, and RAF family, that has an IC50 for WT BRAF at 25 nm [6] and binds to the hydrophobic pocket of the ATP binding cleft to indirectly compete with ATP. The structure of the fusion protein readily suggests that sorafenib may also inhibit activity of *KIAA1549-BRAF* as the *BRAF* kinase domain is completely conserved. Interestingly successful targeting by sorafenib of BRAF-fusion bearing cells specifically the *AGK-BRAF* fusion that contains the first 33 amino acids of AGK and the C-terminal portion of BRAF starting from position 328 of BRAF with loss of the CR1 domain (not *KIAA1549-BRAF*) has been recently reported [7]. In the experiments carried out the patient-derived *AGK-BRAF* expressing melanoma cell line demonstrated an increased sensitivity to sorafenib [7]. Remarkably, sorafenib was also active against *RAF* fusion proteins reported in prostate cancer [8].

Notably, pediatric astrocytoma patients expressing this fusion progressed on sorafenib monotherapy in a small phase II trial [9]. This observation suggests that sole inhibition of the *BRAF* fusion is insufficient for tumor response. An analogy can be made to non-melanoma solid tumors expressing the activating V600E mutant *BRAF*, where vemurafenib treatment is sometimes ineffective, ie as in the colon [10]. In such instances of resistance aberrant activation of the P13K/PTEN/mTOR pathway has been implicated [10-12]. This leads to the premise that combined BRAF and mTOR pathway inhibition may overcome the innate and/or acquired resistance [13-16] to BRAF-target monotherapy, as has been demonstrated in other systems [17]. Thus, the combination therapy administered in this patient seemingly targeted the existing genomic alterations. Interestingly, in murine models of pilocytic astrocytoma, the *KIAA1549-BRAF* fusion hyperactivates the mTOR pathway [18], as does loss of *PTEN *[18]. In this context, the success of this combination therapy suggests both inhibition of the *BRAF* fusion protein by sorafenib and of the downstream mTOR pathway by temsirolimus synergizes, although the specific mechanism of tumor regression awaits further investigation. Bevacizumab was also administered, but the contribution of this therapy to the patient response is difficult to ascertain. As has been theorized for other tumor types, it is possible that bevacizumab enhanced delivery of the other therapeutics by normalizing the tumoral vasculature [19].

What are the implications of this case for treating tumors that express the *KIAA1549-BRAF* fusion such as pediatric pilocytic astrocytomas?

As patients with such tumors on sorafenib monotherapy progressed rapidly in a very small Phase II study [9], sorafenib combination therapy combined with mTOR inhibitors is an alternative avenue. While this is suggestive, additional *in vitro* and *in vivo* studies well outside the scope of this report are required. Sorafenib is indeed not a specific inhibitor of BRAF and as such the effects may not be mediated by targeting *KIAA1549-BRAF* in this tumor.

Specifically, genomic profiling based on clinical-grade NGS could identify whether *PTEN* loss or other GAs activating the mTOR pathway are present alongside the *BRAF* fusion in a pilocytic astrocytoma of patient, thus suggesting a potential responsiveness to combined sorafenib/mTOR targeted therapy. As a possible therapeutic alternative, a recent *in vitro* study suggested that second generation BRAF inhibitors may be capable of effectively inhibiting this fusion protein [20].

## Conclusions

A prospective clinical trial with prospective diagnostic genomic profiling will best answer whether tumors driven by *KIAA1549-BRAF* fusions typically coincide with alterations in the mTOR pathway, and whether such patients can be effectively treated with targeted therapy on that basis. However, this case already indicates such treatment possibilities exist for patients possessing *KIAA1549-BRAF* fusions, whether in pediatric astrocytomas or difficult to characterize sarcoma like lesions. In general, genomic profiling based on NGS may be of great relevance in the management of patient with solid tumors that defy pathologic definition, as this case demonstrates that such diagnostics can reveal unexpected but effective avenues of targeted treatment.

## Findings

• *KIAA1549-BRAF* fusion, a recurrent oncogenic driver in sporadic pilocytic astrocytoma, is described here in a spindle cell neoplasm for the first time.

• This case is the first report any tumor expressing *KIAA1549-BRAF* fusion with *PTEN* loss to be successfully treated by targeted therapy.

• Genomic profiling based on Next-generation sequencing (NGS) may be of great relevance in the management of patient with solid tumors that defy pathologic definition, as this case demonstrates that such diagnostics can reveal unexpected but effective avenues of targeted treatment.

## Competing interests

KW, SMA, VAM, PJS, GAP, and JSR are all employees of and own stock in Foundation Medicine.

## Authors’ contributions

All authors contributed to writing the manuscript. VS, VAM and SMA conceived the manuscript. VS and DA provided clinical expertise. SNW is the primary investigator for the clinical trial and VS is the co-primary investigator for the clinical trial. VS, SNW, KW, WW, VAM, JR, PJS, GAP and SMA analyzed the data. WW, JR, SMA provided pathology expertise. VS and SMA wrote the paper. VS and DA provided sarcoma expertise. VS and SMA provided cellular, molecular and targeted therapy expertise. All authors read and approved the final manuscript.
